# Baduanjin exercise in the treatment of hypertension: A systematic review and meta-analysis

**DOI:** 10.3389/fcvm.2022.936018

**Published:** 2022-08-15

**Authors:** Zhen Ma, Honghui Lei, Kexin Tian, ZhiZe Liu, Ying Chen, Haoqi Yang, Xiangyu Zhu

**Affiliations:** ^1^Department of Rehabilitation, School of Acupuncture-Moxibustion and Tuina, Beijing University of Chinese Medicine, Beijing, China; ^2^Department of Encephalopathy, Beijing Dongzhimen Hospital, Beijing University of Chinese Medicine, Beijing, China; ^3^Department of Sports Rehabilitation, School of Kinesiology, Shanghai University of Sport, Shanghai, China

**Keywords:** Baduanjin, hypertension, BP, SBP, DBP, exercise

## Abstract

**Background:**

As a therapy to prevent and treat hypertension, exercise is widely used in clinical practice. But due to the lack of documentary evidence, Baduanjin as a relaxed and convenient mode of exercise is not currently recommended by professional health organizations to treat hypertension. The purpose of this article is to examine the efficacy of Baduanjin as an antihypertensive exercise therapy.

**Methods:**

Our systematic retrieved of the entire relevant literatures in 12 databases. Finally, 28 eligible trials involving Baduanjin intervention in hypertension were included. After the quality assessment and bias risk assessment of the included trials, we analyzed the blood pressure values before and after the intervention, and performed meta-analysis on the random effect results. In order to explore the factors influencing the decrease of blood pressure, we also performed a subgroup analysis of the results.

**Results:**

Participants (*n* = 2121) were adults (61.74 ± 5.85years of age, mean ± SD), with baseline blood pressure (systolic blood pressure (SBP) = 150.7 ± 9.2 mmHg, diastolic blood pressure (DBP) = 93.2 ± 8.8 mmHg). Baduanjin was practiced 7.5 ± 3.8 sessions / week for 28.2 ± 12.8 min /session for 16.7 ± 9.2 weeks. Overall, Baduanjin resulted in SBP (−9.3 mmHg, d = −1.49, 95%CI: −1.73 to −1.13) and DBP (−6.3 mmHg, d = −1.20, 95%CI: −1.51 to −0.88) vs. the control group (*p* < 0.001). After a subgroup analysis of age, we found that SBP heterogeneity was significantly reduced in the elderly group.

**Conclusion:**

Our results indicate that Baduanjin can effectively reduce blood pressure (i.e., 9.3 mmHg and 6.3 mmHg of SBP and DBP reductions, respectively), and reduce the incidence rate of cardiovascular disease in hypertensive patients. In addition, we will be more likely to recommend that the elderly exercise Baduanjin.

## Introduction

Hypertension is a preventable risk factor of cardiovascular disease, which is closely linked to people's lifestyle. Both aerobic exercise (1) and resistance exercise (2) have been demonstrated to effectively reduce blood pressure. Through regular aerobic exercise, blood pressure (BP) can be typically decreased overall by 5 mmHg (3), the mortality of coronary heart disease (CHD) reduces 9%, the mortality of stroke reduces 14% and all-cause mortality also reduces 7% (4). Therefore, compared to using drug which may cause side effects, regular moderate- and low-intensity aerobic training should be more recommended for hypertensive patients and healthy adults. Nevertheless, due to the high requirements of aerobic exercise and resistance training on physical strength and endurance, most adults with high blood pressure are unwilling or unable to adhere to exercise, thus choose safer, less strenuous forms of exercise like Taijiquan, yoga and Baduanjin. However, compared with other forms of movement, Baduanjin is simply, convenient, inexpensive, serviceable (5), less time in practice and easier to learn. Therefore, Baduanjin is more suitable for those adults who are reluctant to exercise than Taijiquan and yoga.

Baduanjin pays attention to the rhythm coordination of breathing and movement, and improves the morphology and physiological function of the human body (1, 2). Its curative effects include improving the strength of the upper and lower limbs, promoting the cardiovascular function, promoting the respiratory system function, improving the joint flexibility, improving the balance ability and the sensibility of the middle-aged and elderly people and so on (6). Among them, the antihypertensive effect in adult patients with hypertension is one of the most commonly used curative effects in clinic (7, 8). However, due to the lack of documentary evidence, professional associations and organizations, such as the International Society of Hypertension (9), do not currently recommend Baduanjin as a non-pharmacological treatment equivalent to aerobic exercise.

Three meta-analyses currently published in English [10 to 12] had quantified the BP-lowering effect of Baduanjin, one of the meta-analyses for essential hypertension reported a standardized mean difference (SMD) of 1.80 mmHg for systolic blood pressure (SBP) and 0.22 mmHg for diastolic blood pressure (DBP) (12). A meta-analysis reported a clinical non-standardized mean difference MD of 8.52 mmHg for SBP and 4.65 mmHg for DBP (11). The 3rd meta-analysis used weighted mean differences (WMD) to quantify the resulting of 13.00 mmHg for SBP and 6.13 mmHg for DBP (10). Despite these quantitative results are meaningful, these meta-analyses have the following limitations: (a) The sample size for all meta-analyses was very small (k = 8 to 14; *n* = 572–1058), and only 6 to 8 databases were retrieved. (b) Heterogeneity was high (10, 11) (I^2^ ranged from 90 to 93%), but none of the analysis reduced the heterogeneity was performed.

To address the limitations in the above meta-analysis, we conducted a meta-analysis of the largest sample to examine the efficacy of Baduanjin in reducing blood pressure, and to explore the antihypertensive mechanism of Baduanjin. In addition, we performed a subgroup analysis to investigate the causes affecting the changes in blood pressure to provide ideas for the clinical prevention and treatment of hypertension through exercise.

## Methods

### Selection criteria

The protocol of this systematic evaluation and meta-analysis procedures was registered in PROSPERO (registration number: CRD42021291953), and was conducted in accordance with the Preferred Reporting Items for Systematic Reviews and Meta-analyses Statement (13). According to seven inclusion criteria, the trials were eligible if the following conditions were observed: (a) adults older than 18. (b) there were no more physical diseases except for hypertension. (c) stating the intervention was Baduanjin. (d) the participant's health could support at least 4weeks of Baduanjin exercise. (e) including the non-exercise / non-dietary controls. (f) trials included BP reports pre- and post-intervention the intervention in Baduanjin and control groups and (g) all trials were peer-reviewed articles published in either English or Chinese language journals. Trials were excluded if they (a) were literature reviews, case-control trials, or animal research literature. (b) had repeated publications, unclear outcome measures, or obvious data errors. (c) included fewer than 10 samples. (d) included samples of other diseases (e.g., cardiovascular disease, diabetes). (e) involved a single Baduanjin session (i.e., an acute intervention).

### Search strategy

We systematically searched all articles in 8 English electronic databases and 4 Chinese electronic databases (Supplementary Table 1, the complete search strategy for all queried electronic databases). Team coders screened the report twice, first with the title and abstract, and then with the full text. Additional reports were also searched in the reference lists of included trials, related reviews and meta-analysis.

### Data extraction

We extracted the SBP and DBP as outcome indicators. We also extracted some basic characteristics that might effect a change in blood pressure, such as the mean age of trials, intervention studies, exercise intensity, teacher qualifications, medications, dietary maintenance and exercise habits. In the collection of resting BP, we used the BP value before the start of the Baduanjin intervention as the baseline BP (pre-intervention value) (14), used the BP value taken at the time point after and closest to the end of the Baduanjin intervention as the outcome indicator (post-intervention value). Since most of the trials in this literature were conducted prior to the publication of the 2020 ISH guidelines, we followed the 2020 International Society of Hypertension Global Hypertension Practice Guidelines definitions of hypertension and normotensive in our meta-analysis (9).

The Cochrane Risk-of-Bias Tool for Randomized Trials (ROB 1.0) (15) was used to evaluate the quality of methodological research, including random sequence generation, allocation concealment, blinding of participants and personnel, blinding of outcome assessment, incomplete outcome data, selective reporting (RCT) and other bias.

Furthermore, we used the Methodological Index for Non-randomized Studies (MINORS) (16) to assess risk of bias in various areas, including clearly gave the research purpose, the coherence of the participants, expected data collection, the end index can properly reflect the objective, objectivity of the endpoint index evaluation, adequate follow-up time, follow-up rate is <5%, whether the sample size is estimated, whether the choice of the control group is appropriate, whether the control group is synchronized, whether the baseline is comparable among the groups, and whether the statistical analysis is appropriate (NRCTs). There were 12 evaluation indicators, each one could be scored from 0 to 2 points, the highest score was 24 points, 0 to 8 was low quality, 9 to 16 was medium quality, and 17 to 24 was high quality.

All data extractions and risk of bias assessment were carried out by teams of assessors working in pairs, and any uncertainties or disagreements were resolved by involving a third assessor, and all differences were resolved through discussion and high reliability was achieved.

### Outcome and effect size calculations

Our meta-analysis was conducted to explore the antihypertensive effect of Baduanjin. Since we observed large differences in the distribution of standard deviations of baseline blood pressure in the included trials SBP (the SD ranged from 1.7 to 12.6) and DBP (the SD ranged from 0.9 to 10.8), we chose to use standardized effect sizes. Which were more robust and less biased, and have better effective compared to unstandardized effect sizes across many statistical circumstances (17).

### Publication bias

We evaluated the potential publication bias and other reporting bias of SBP and DBP by visually observing the symmetry and distribution of funnel plots, used the tests of Begg and egger et al (18).

### Sensitivity analysis

Notably, we included all RCT and nRCT samples. In fact, RCT (92.9%, k = 26) was much more than nRCT (7.1%, k = 2). Therefore, a sensitivity analysis was used to compare the SMD values for all trials (including RCT and nRCT) and the RCT-only trials (19). The mean effect sizes showed no difference between experiments that contained both RCT and nRCT and experiments that contained RCT-only, confirming the decision to combine RCT and nRCT for analysis.

### Statistical calculation

The analysis was performed using the Stata15 version (20), and the Stata command appeared in Supplementary Table 2 (Stata command). Descriptive statistics were reported as mean ± standard deviations unless noted otherwise. Two-sided significance level was *p* < 0.05.

## Results

### Study search and study characteristics

**Figure 1:**
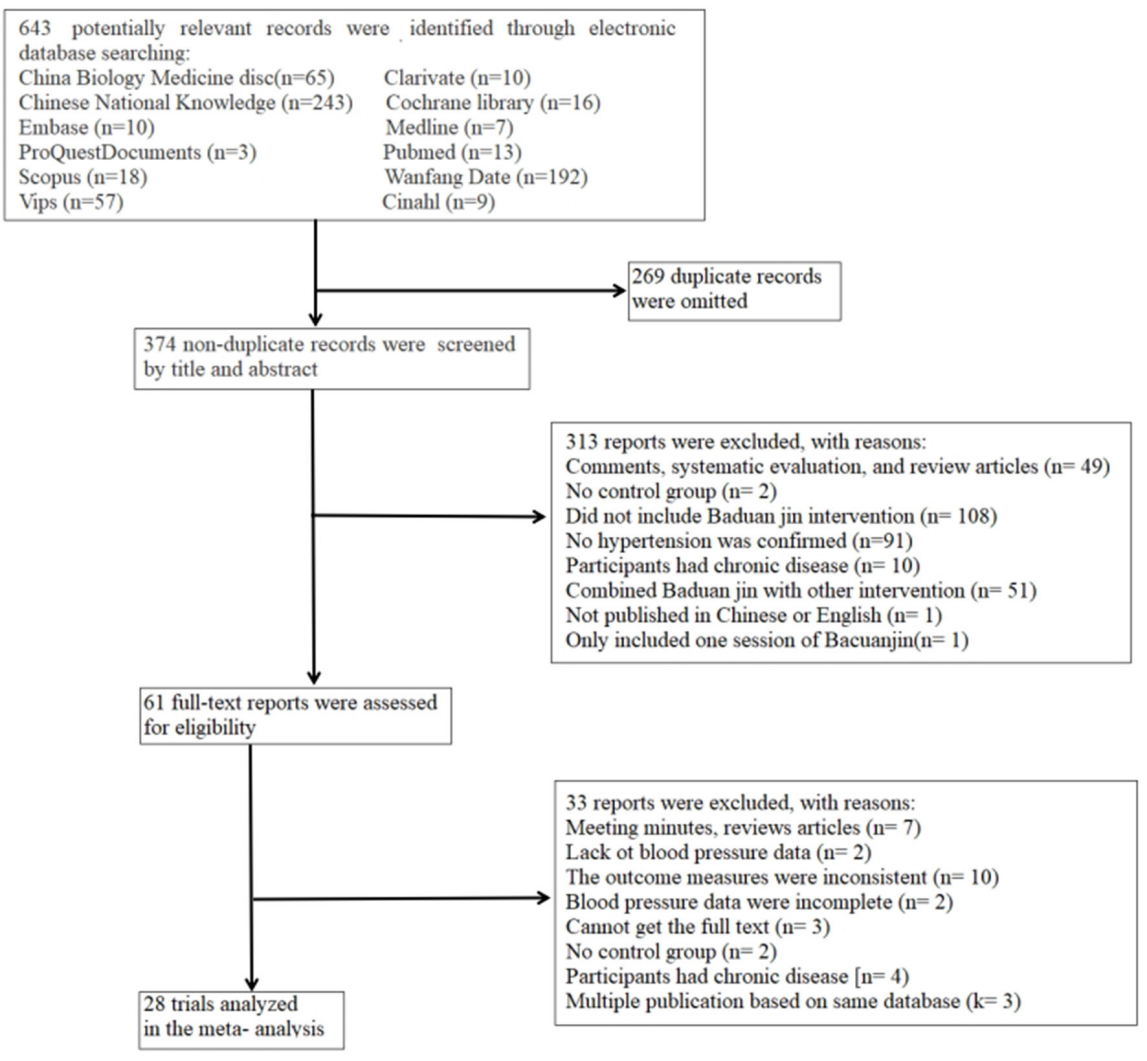
Flow chart detailing the systematic search of potential reports and selection process of included Baduanjin trials.

We finally included 28 Baduanjin controlled trials that met the inclusion criteria (Figure 1). A detailed list of included trials was shown in Supplementary Table 3 (reference list for the included trials).

The Baduanjin intervention trials were published before 2021, including RCTs (k = 26, 92.9%) or NRCTs (k = 2, 7.1%), and all trials use BP as the primary outcome. Among them, 25 trials (89.3%) disclosed the intervention measures of the control group (Supplementary Table 4, main characteristics of the Baduanjin intervention), including activities of daily living (k = 7, 25.0%), daily care (k = 2, 7.1%), health education (k = 14, 50.0%) and daily walking (k = 2, 7.1%). The remaining 3 (10.7%) control groups did not disclose the intervention measures. All of these trials were published in English (k = 1, 3.6%) and Chinese (k = 27, 96.4%) language journals.

The risk of bias score for individual trials was shown in Supplementary Table 5 (Risk of bias for RCTs) and Supplementary Table 6 (Risk of bias for NRCTs). Based on ROB1.0, the majority of RCTs (k = 12, 80%) appeared high levels of risk of bias. Especially, blinding of outcome assessment was rated as the area with the highest risk of bias (k = 24, 92.3%). Meanwhile, nRCTs (k = 2,100%) appeared lower risk of bias based on MINORS, and objectivity of the endpoint index evaluation was rated as the area with a higher risk of bias proportion of trials with risk (k = 2,100%).

### Sample characteristics

The baseline sample characteristics of Baduanjin (*n* = 1061) group and control group (*n* = 1060) were similar (*p* > 0.05) (Supplementary Table 7 baseline sample characteristics). On average, participants (*n* = 2121) were adults of all ages with hypertension (SBP = 150.7 ± 9.2mmHg / DBP = 93.2 ± 8.8 mmHg), mostly of whom were older adults (61.8 ± 6.0 years), and the proportion of male and female participating was proximity, including the proportion of female (49.91 ± 7.81%). In the included sample, the baseline pulse pressure (PP) was 57.54 ± 8.47 mmHg, and the different value of pulse pressure was 5.69 ± 4.24 mmHg between a comparison pre-and post-Intervention. A total of 17 trials (60.7%) explicitly reported regarding medication use. Among them, 9 trials reported specific information on the blood pressure drugs used, and Amlodipine (a calcium channel blocker) was the most popular (7 trials reported). Three trials were reported using the Micardis (telmisartan), Lercanidipine, Thiazide diuretics, losartan potassium. Nifedipine and traditional Chinese medicine had been reported by one trial respectively.

We also summarized the characteristics of Baduanjin intervention. On average, Baduanjin was practiced 7.5 ± 3.8 sessions / week for 28.2 ± 12.8 min / session for 16.7 ± 9.2 weeks. Among the trials, eight of them explicitly appeared that the daily workouts were once in the morning and once in the afternoon. In addition, there are two styles of Baduanjin interventions included sitting Baduanjin (k = 3, 10.7%) and standing Baduanjin (k = 25, 89.2%).

All trials (k = 28, 100.0%) supervised and instructed participants, but less than half of the trials disclosed the information of teachers (k = 11, 39.3%), including Traditional Chinese Medicine (TCM) professionals (k = 9, 32.1%) and professional certificates (k = 2, 7.1%). Two trials disclosed the number of teachers involved in the training. In addition, a few parts of the trials used video-assisted to instruct Baduanjin (k = 4, 14.3%). It is worth noting that all of these 4 trials took the method of punch the clock on the Internet, in order to urge the enthusiasm for and sustainability of Baduanjin training. Only one trial emphasized the attention to breathing skills and the consistency and stability of the movement (21). Finally, in our sample, 2 trials (3.2%) indicated that some participants withdrew from the study due to loss to follow-up. The remaining trials (k = 26, 92.9%) did not provide any information on adverse events.

### Resting BP assessment

Most trials (k = 16, 57.1%) reported the use of BP measurement instruments, including automatic/digital sphygmomanometers (k = 3, 10.7%) or manual sphygmomanometers (k = 13, 46.4%). A portion of the trials (k = 7, 25.0%) reported a sitting posture during BP measurement. Six trials (k = 6, 21.4%) reported that blood pressure was measured between 8 a.m. and 10 a.m. at a fixed time. However, one-quarter of the trials (k = 8, 28.6%) did not report any details of the resting blood pressure measurement procedures.

### Baduanjin as stand-alone antihypertensive therapy and potential mechanisms

**Figure 2:**
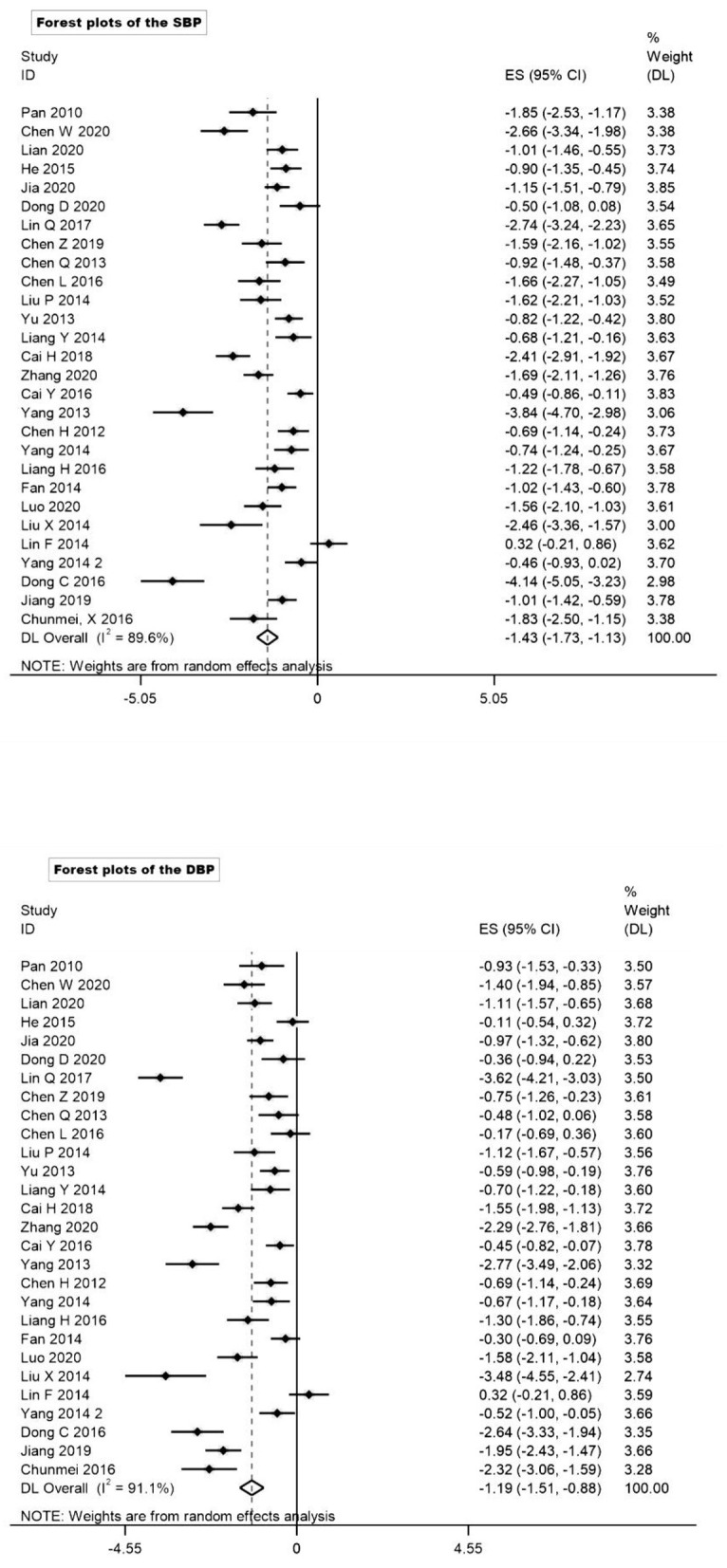
Age subgroup analysis of SBP and DBP.

Due to the high heterogeneity of SBP (I^2^ = 89.6% > 50%, 95%CI: 86.18–92.20%, *P* < 0.001) and DBP (I^2^ = 91.1 > 50%, 95%CI: 88.28%−93.21%, *P* < 0.001), the standardized effect sizes lacked homogeneity. Therefore, we selected random effects meta to analysis. Overall, compared with the control group (*p* < 0.001), there was significant reduction in SBP (to 9.3 mmHg, SMD = −1.49, 95%CI: −1.73 to −1.13) and DBP (to 6.3 mmHg, SMD = −1.20, 95%CI: −1.51 to −0.88) in the Baduanjin group (Figure 2, Forest plots of the SBP and DBP response to Baduanjin vs. control). Furthermore, for SBP (to 1.49 vs. −1.38) and DBP (to 1.20 vs. −1.19), the mean effect size contained both RCT and nRCT, which was similar to the mean effect size that contained RCT to only. This supported our decision to combine RCT to nRCT in the final analysis.

Regarding the mechanisms of the hypotensive effect of Baduanjin (Supplementary Table 4), 17 trials (60.7%) discussed the potential mechanisms of hypotensive. However, only 5 trials (21.4%) attempted to verify the mechanism discussed. Inside, 3 trials (10.7%) examined the relationship between the BP changes and the measurement mechanisms (22–24). Lin Q compared serum C to reactive protein (CRP) and found that Baduanjin could inhibit the secretion of inflammatory cytokine CRP, reduce the expression level of serum CRP, and actively promote the control of blood pressure. Chen Z considered that Baduanjin exercise could improve Nitric Oxide Synthase (NOS) expression and promote Nitric Oxide (NO) synthesis. Chunmei considered that Baduanjin decreased the level of Endothelin to 1 **(**ET to 1) in skeletal muscle plasma and increased serum NO in patients with essential hypertension.

### Subgroup analysis

In the process of data extraction, we found that some trials explicitly defined participants as the aged, but others did not have a strict age limit (18–70 years). Therefore, in order to explain the exercise effect of the elderly patients after Baduanjin exercise, we divided the included trials into the elderly group (group 1, clearly defined participants as the elderly or defined participants ≥ 60 years old) and the adult group (group 2).

**Figure 3:**
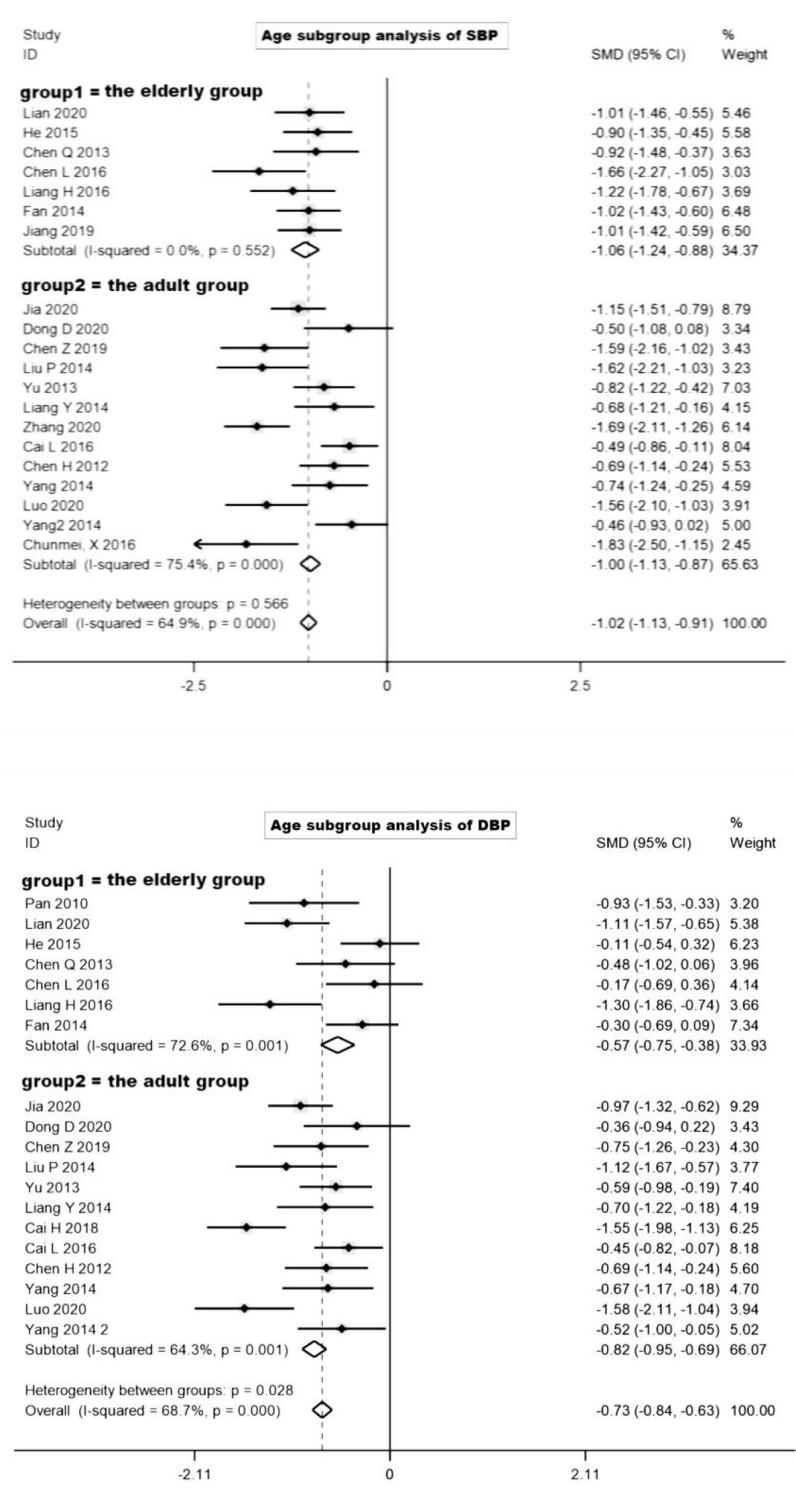
Subgroup analysis of Baduanjin types.

In the age subgroup analysis of SBP (Figure 3, Age subgroup analysis of SBP and DBP), after the removal of three trials that greatly affected the heterogeneity (possibly affected by the study characteristics, sample characteristics) (25–27), seven trials were included in the elderly group. Moreover, the heterogeneity of the elderly group was significantly reduced (I^2^ = 0.0%). The results displayed that Baduanjin significantly reduced SBP compared with the control group, and SBP was statistically significant (to 11.2 mmHg, SMD = −1.06, 95%CI: −1.24 to −0.88). In the adult group (I^2^ = 75.4%), thirteen trials were included after removing five trials that greatly affected the heterogeneity. Compared with the control group, blood pressure decreased significantly, and SBP was statistically significant (to 8.2 mmHg, SMD = −1.00, 95%CI: −1.13 to −0.87).

In the age subgroup analysis of DBP, seven trials were included when the elderly group excluded three trials (26–28) with a great impact on heterogeneity. Meantime, the heterogeneity of the elderly group decreased (I^2^ = 72.6%). Compared with the control group, Baduanjin reduced blood pressure significantly, and the difference value of DBP was statistically significant (to 3.3 mmHg, SMD = −0.57, 95%CI: −0.75 to −0.38). In the adult group (I^2^ = 91.4%), twelve trials were included after removing six trials that greatly affected the heterogeneity. Meantime, blood pressure reduction was significant compared with the control group, and DBP difference value was significant (to 4.7 mmHg, SMD = −0.82, 95%CI: −0.95 to −0.69).

**Figure 4:**
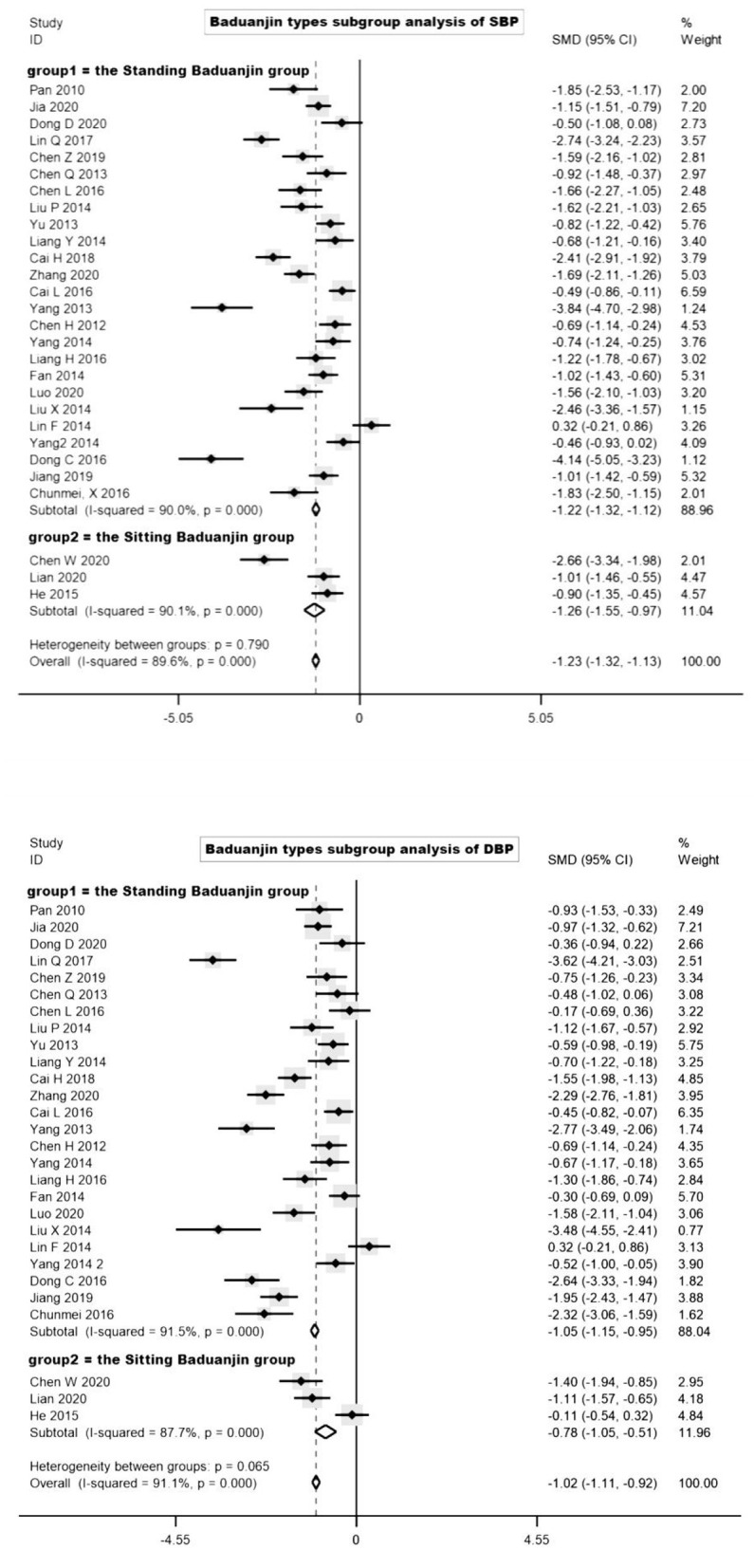
Subgroup analysis of Baduanjin types.

We also conducted a subgroup analysis for different types of Baduanjin (29) (Figure 4, subgroup analysis of Baduanjin types). The included trials were divided into the Standing Baduanjin group (group 1) and the Sitting Baduanjin group (group 2) based on different exercise movements, and these two groups included 25 trials and 3 trials respectively. Compared with the control group, the Sitting Baduanjin group (I^2^ = 90.1%) could significantly reduce SBP (to 12.8 mmHg, SMD = −1.26, 95%CI: −1.26 **to –**0.97), and the difference value was statistically significant. In the Standing Baduanjin group (I^2^ = 90.0%), BP reduction ability was statistically significant compared with the control group, and DBP difference value was statistically significant (to 7.1 mmHg, SMD = −1.22, 95%CI: −1.32 to 1.12). Compared with the control group, the Sitting Baduanjin group (I^2^ = 91.5%) could significantly reduce DBP (to 7.8 mmHg, SMD = −1.02, 95%CI: −1.11 to −0.92), and the difference value was statistically significant. The standing Baduanjin group (I^2^ = 87.7%) also significantly reduced the DBP (to 6.1 mmHg, SMD = −1.05, 95%CI: −1.15 to– 0.95) and the difference value was statistically significant.

Based on the above analysis, there was no heterogeneity in SBP reduction after Baduanjin intervention in the elderly group, and the heterogeneity of DBP was also relatively reduced since performing the subgroup analysis. The effect of BP reduction was statistically significant in both groups. Notably, the decrease of PP in the elderly group (PP = 8.91 ± 4.03 mmHg) was much greater than that in the adult group (PP = 3.90 ± 3.08 mmHg).

### Publication bias

Funnel plots, test of Begg and test of Egger all suggested significant publication bias and other reporting bias in the SBP Baduanjin intervention (test of Begg: z = −3.16, *p* = 0.002; test of Egger: t = −3.68, *p* = 0.001). For the elderly group, publication bias was significantly reduced after excluding three trials with great impact on heterogeneity (test of Egger: *P* = 0.095 > 0.05). For the adult group, the test of Egger's (*P* = 0.249 > 0.005) showed no significant publication bias after excluding five trials that had a great impact on heterogeneity. These results indicated that meta to analysis was robust. (Supplementary Figure 1, Funnel plot of SBP reduction in age subgroups)

Meanwhile, the funnel plot, test of Begg and test of Egger all showed a significant publication bias and other reporting bias in the DBP response to Baduanjin (test of Begg: z = −2.41, *p* = 0.016; test of Egger: t = −3.06, *p* = 0.005). For the elderly group, the test of Egger's (*P* = 0.252 > 0.005) showed no significant publication bias after excluding three trials that had a great impact on heterogeneity. For the adult group, publication bias was significantly reduced after excluding six trials with great impact on heterogeneity (test of Egger: *P* = 0.806 > 0.05), indicating that the analysis result was robust (Supplementary Figure 2, Funnel plot of SBP reduction in age subgroups)

## Discussion

Our meta to analysis examined the efficacy of Baduanjin as an exercise therapy to reduce blood pressure, which is the largest sample size examined to date. We found that Baduanjin exercised an average of 7.5 ± 3.8 sessions / week for 28.2 ± 12.8 min /session for 16.7 ± 9.2 weeks, produced SBP reductions of 9.3 mmHg and DBP reductions of 6.3 mmHg. Since performing the subgroup analysis, our finding was that Baduanjin has low heterogeneity in reducing blood pressure in the elderly group. This may indicated that compared with adults, the curative effectiveness of Baduanjin training in elderly patients with hypertension was more controllable. In the elderly group, the Baduanjin intervention reduced 8.7 mmHg and 4.8 mmHg of SBP and DBP respectively. The reduction in blood pressure reduces the risk of heart disease by about 22% and the stroke risk by about 29% (30). These large BP reductions support the use of Baduanjin as a safe and effective exercise method to reduce patients' BP in daily life. In particular, it is recommended that Baduanjin be used as a lifestyle for the prevention and treatment of hypertension among the elderly.

Regular aerobic exercise can lower BP, thereby reducing the risk of cardiovascular disease, hence it is a recommended exercise mode for many medical organizations around the world to prevent and treat hypertension. However, due to the high physical fitness requirements of aerobic exercise, most adults with hypertension are unwilling or unable to exercise. Therefore, in recent years, many alternative therapies for aerobic exercise, such as Taijiquan (31–33) and yoga (34–36), have been widely recommended in clinical practice. But after comparing with other exercises beneficial to blood pressure, we also recommend Baduanjin exercise as a way to treat hypertension in daily life. Yin Wu believed that Taijiquan elicited SBP reductions of 11.3 mmHg and DBP reductions of 4.8 mmHg (31), yoga elicited SBP reductions of 5.0 mmHg and DBP reductions of 3.9 mmHg (34). We found that Baduanjin elicited SBP reductions of 9.3 mmHg and DBP reductions of 6.3 mmHg. When the curative effects are similar, Baduanjin is safer, more convenient, simpler and easier to learn than Taijiquan and yoga. A complete set of Baduanjin exercise plus warm to up and stretching take < 20 min, and do not need other auxiliary required. It is more helpful to those adults who are not willing to exercise keep exercising. In addition, Baduanjin is also widely used in the prevention, treatment and rehabilitation research of nervous system diseases (stroke, etc.), respiratory system diseases (chronic obstructive pneumonia, COVID to 19, etc.), cardiovascular system diseases (coronary heart disease, etc.) and musculoskeletal system diseases. It can improve balance function, regulate blood glucose, regulate negative emotions, and improve cognitive function (37–40).

Our results showed that if the participants were limited to the elderly, the effect of reducing blood pressure after Baduanjin intervention was reliable. With the increase of age, a series of physiological changes will occur in the human body, such as increased arterial stiffness, elevated PP, changed in the sensitivity and function of the autonomic nervous system, changed in the endothelial function and so on (41). In fact, PP and arterial stiffness are significant predictors of morbidity and mortality in the elderly (42). Every 10 mmHg increases in PP increases the risk of cardiac insufficiency 32% and the risk of stroke 24% (43). Therefore, reducing the pulse pressure D to value can reduce the risk of atherosclerosis and cardiovascular disease. Our study observed that the decrease of PP in the elderly group (PP = 8.91 ± 4.03 mmHg) was much greater than that in the adult group (PP = 3.90 ± 3.08 mmHg). It proved the view that Baduanjin could prevent arteriosclerosis in the elderly.

We also tried to group according to different types of Baduanjin. Sitting Baduanjin (k = 3, 10.7%) has gentle movement and a small range of activities, focusing on the cultivation of Chinese Taoist "Qi.” Standing Baduanjin (k = 25, 89.2%) has forceful movement and a large range of activities, which is more like a kind of martial art (44). However, our results showed that the heterogeneity within and between groups were very high according to the different types of Baduanjin. The reasons for this result probable were that (a) the trials used sitting Baduanjin were rare. (b) there were also many differences categories in Standing Baduanjin. Therefore, based on the current trials, it is unable to classify according to the type of Baduanjin.

Due to space constraints or other unknown reasons, it was still possible that the included Baduanjin trials were inadvertently missing some study details. This limited our ability to extract information and analysis it. Therefore, we were unable to analyze the source of factors effecting blood pressure reduction, such as teachers' qualifications (39.3% reported), whether to emphasize the skills of Baduanjin practice (3.6% reported), blood pressure measurement methods (57.1% reported), baseline medication (60.7% reported) and medication during the intervention (60.7% reported), dietary maintenance (30.2% reported) and physical activity level (10.7% reported).

We recommend that readers make appropriate reference to our research results. First, all the included trials were evaluated using ROB1.0 and MINORS, most of them had high / serious bias risks. Second, almost all the participants in the trials were Chinese, lacking research samples from other countries.

Our meta to analysis also had several strengths. First, our research covered a wide range Baduanjin types. Second, we focused on the effectiveness of Baduanjin on the blood pressure of the elderly, which had certain reference value.

## Conclusion

Our noteworthy analysis results are that Baduanjin interventions in the elderly group elicited SBP reductions of about 11.2 mmHg and DBP reductions of about 3.3 mmHg. Baduanjin may be another feasible choice for the treatment of hypertension, and can be appropriately popularized in the elderly. It can reduce the PP of the elderly and prevent the occurrence of cardiovascular disease. The current review also calls attention to the fact that future Baduanjin intervention studies need to (a) pay more attention to the research on the hypotensive mechanism of Baduanjin. (b) study the exercise effect of different age groups, and strictly distinguish age grades. (c) study the difference of curative effectiveness of different types of Baduanjin.

## Data availability statement

The datasets presented in this study can be found in online repositories. The names of the repository/repositories and accession number(s) can be found in the article/Supplementary material.

## Author contributions

ZM and XZ participated in the design of the study. HL, ZM, and KT contributed to data analysis and interpretation. ZL, YC, ZM, and HY contributed to data collection. XZ, HL, ZM, and KT contributed to data interpretation. All authors contributed to manuscript writing, agree with the order of presentation of the authors, read, and approved the final version of the manuscript.

## Funding

This study was funded by a grant from the National Natural Science Foundation of China (No. 82105034).

## Conflict of interest

The authors declare that the research was conducted in the absence of any commercial or financial relationships that could be construed as a potential conflict of interest.

## Publisher's note

All claims expressed in this article are solely those of the authors and do not necessarily represent those of their affiliated organizations, or those of the publisher, the editors and the reviewers. Any product that may be evaluated in this article, or claim that may be made by its manufacturer, is not guaranteed or endorsed by the publisher.
