# Supplementary material for: Baduanjin exercise in the treatment of hypertension: A systematic review and meta-analysis
Source: Front Cardiovasc Med. 2022 Aug 15;9:936018. doi: 10.3389/fcvm.2022.936018 (PMC9421065; doi:10.3389/fcvm.2022.936018)

Supplementary Material

# Supplementary Table 1.Full search strategy for each of the electronic databases queried.

| **search strategy** | | | |
| --- | --- | --- | --- |
| **Table1**.CNKI(China National Knowledge Infrastructure) | | | |
| **Number** | **Search Terms** | **Search String** | **Hits:** |
| 1 | Baduanjin | （（主题％＝＇八段锦＇or题名％＝＇八段锦＇or title=xls'八段锦＇）orv  subject=xls('八段锦＇）） |  |
| 2 | Hypertension | ((((主题％＝＇高血压＇or题  名％＝＇高血压＇or title=xs ('高血压＇） or v subject=xls ('高血压＇））OR(主题％＝＇心血管疾病＇or 题名％＝心血管疾病＇or  title=xls (心血管疾病＇） orv subject=xls('心血管疾  病）））OR(主题％＝心血管＇or题名％＝＇心血管＇or title=xls ('心血  管＇） orv subject=xs ('心血管＇）））OR(主题％＝＇血压＇or 题  名％＝＇血压＇or title=xls ('血压） orv su bject=xls (血压＇）））） |  |
| 3 | Combined search | #1 AND #2 |  |
| 4` | Date | inception 1999 – January 31st, 2021 | **243** |
| Note* translation: 主题=subject; 题名=title; 八段锦=baduanjin; 高血压=hypertension; 心血管= cardiovascular;  心血管疾病= angiocardiopathy; 血压= blood pressure | | | |
|  | | | |
| **Table2.** VIP | | | |
| **Number** | **Search Terms** | **Search String** | **Hits:** |
| 1 | Hypertension | ((题名或关键词=高血压 OR 题名或关键词=血压) OR 题名或关键词=心血管疾病) |  |
| 2 | Baduanjin | 题名或关键词=八段锦 |  |
| 3 | Combined search | #1 AND #2 |  |
| 4 | Date | inception 1949 – January 31st, 2021 | **57** |
| Note* translation: 题名或关键词=title or key word; | | | |
|  | | | |
| **Table3**.Wanfang | | | |
| **Number** | **Search Terms** | **Search String** | **Hits:** |
| 1 | Hypertension | 主题:(高血压+心血管疾病+血压) |  |
| 2 | Baduanjin | 主题:(八段锦) |  |
| 3 | Combined search | #1 AND #2 |  |
| 4 | Date | inception 1950 – January 31st, 2021 | **192** |
|  | | | |
| **Table4**.CBM(Chinese Biology Medicine) | | | |
| **Number** | **Search Terms** | **Search String** | **Hits:** |
| 1 | Hypertension | ("高血压"[标题:智能] OR "血压"[标题:智能] OR "高血压前期"[标题:智能] OR "心血管疾病"[标题:智能]) |  |
| 2 | Baduanjin | (八段锦) |  |
| 3 | Combined search | #1 AND #2 |  |
| 4 | Date | inception 1978 – January 31st, 2021 | **65** |
| Note* translation: 标题=title;智能=intelligent | | | |
|  | | | |
| **Table5**.Pubmed | | | |
| **Number** | **Search Terms** | **Search String** | **Hits:** |
| 1 | Baduanjin | (((((((Baduanjin[MeSH Terms]) OR (Baduanjin Qigong[Title/Abstract])) OR (eight-section brocades[Title/Abstract])) OR (eight-section exercises[Title/Abstract])) OR (eight-treasured exercises[Title/Abstract])) OR (baduanjin exercise[Title/Abstract])) OR (baduanjin exercises[Title/Abstract])) |  |
| 2 | Hypertension | ((((((((((((((((((((((((((((Hypertension[MeSH Terms]) OR (Cardiovascular Diseases[Title/Abstract])) OR (cardiovascular diseases[Title/Abstract])) OR (High Blood Pressures[Title/Abstract])) OR (High Blood Pressure[Title/Abstract])) OR (Blood Pressures, High[Title/Abstract])) OR (Blood Pressure, High[Title/Abstract])) OR (blood pressure[Title/Abstract])) OR (lood pressures[Title/Abstract])) OR (arterial pressure[Title/Abstract])) OR (arterial pressures[Title/Abstract])) OR (hypotension[Title/Abstract])) OR (normotension[Title/Abstract])) OR (hypertensive[Title/Abstract])) OR (hypotensive[Title/Abstract])) OR (normotensive[Title/Abstract])) OR (systolic pressure[Title/Abstract])) OR (diastolic pressure[Title/Abstract])) OR (pulse pressure[Title/Abstract])) OR (venous pressure[Title/Abstract])) OR (pressure monitor[Title/Abstract])) OR (pre hypertension[Title/Abstract])) OR (bp response[Title/Abstract])) OR (bp reduction[Title/Abstract])) OR (bp monitor[Title/Abstract])) OR (bp decrease[Title/Abstract])) OR (bp monitors[Title/Abstract])) OR (bp measurement[Title/Abstract])) |  |
| 3 | Combined search | #1 AND #2 |  |
| 4 | Date | 1940s – January 31st, 2021 | 13 |
|  |  |  |  |
| **Table6**.Embase | | | |
| **Number** | **Search Terms** | **Search String** | **Hits:** |
| 1 | Baduanjin | ＃6 ＃4 OR #5  ＃5 （＇eight-section brocades':ab,ti OR 'baduanjin qigong':ab,ti OR'eight-section exercises':ab,ti OR 'eight-treasured exercises':ab,tl OR"baduanjin exercise':ab.ti)AND 'baduanjin qigong':ab,ti  #4 'baduanjin'/exp |  |
| 2 | Hypertension | ＃3 ＃1 OR #2  #2 ＇cardiovascular diseases':ab,ti OR'high blood pressures':ab,ti OR 'high blood pressure':ab,ti OR 'blood pressures,high'ab,ti OR 'blood pressure,high':ab,ti  OR 'blood pressure':ab,ti OR 'blood pressures':ab,ti OR 'arterial pressure':ab,ti OR 'arterial pressures':ab,ti OR 'hypotension':ab,ti OR'normotension':ab,ti OR  ＇hypotensive':ab,ti OR 'hypertensive':ab.ti OR 'normotensive':ab,ti OR 'systolic pressure,:ab,ti OR 'diastolic pressureab':ab,ti OR 'pulse pressure':ab,ti OR  ＇venous pressure':ab,ti OR 'pressure monitor':ab,ti OR 'pre hypertension':ab,ti OR "bp response'ab,ti OR 'bp decrease':ab,ti OR 'bp reduction':ab,ti OR'bp  monitorab'ab,ti OR 'bp monitors'ab,ti OR 'bp measurement':ab,ti  #1 'hypertension'/exp |  |
| 3 | Combined search | #3 AND #6 |  |
| 4 | Date | 1960 – January 31st, 2021 | **10** |
|  | | | |
| **Table7**.Cochrane | | | |
| **Number** | **Search Terms** | **Search String** | **Hits:** |
| 1 | Hypertension | #1 Hypertension  #2 (Cardiovascular Diseases):ti,ab,kw OR (cardiovascular diseases):ti,ab,kw OR (High Blood Pressures):ti,ab,kw OR (High Blood Pressure):ti,ab,kw OR (Blood Pressures, High):ti,ab,kw OR (Blood Pressure, High):ti,ab,kw OR (blood pressure):ti,ab,kw OR (blood pressures):ti,ab,kw OR (arterial pressure):ti,ab,kw OR (arterial pressures):ti,ab,kw OR (Hypotension):ti,ab,kw OR (Normotension):ti,ab,kw OR (Hypertensive):ti,ab,kw OR (Hypotensive):ti,ab,kw OR (Normotensive):ti,ab,kw OR (systolic pressure):ti,ab,kw OR (diastolic pressure):ti,ab,kw OR (pulse pressure):ti,ab,kw OR (venous pressure):ti,ab,kw OR (pressure monitor):ti,ab,kw OR (pre hypertension):ti,ab,kw OR (bp response):ti,ab,kw OR (bp decrease):ti,ab,kw OR (bp reduction):ti,ab,kw OR (bp monitor):ti,ab,kw OR (bp monitors):ti,ab,kw OR (bp measurement):ti,ab,kw OR  #3 #1 OR #2 |  |
| 2 | Baduanjin | #4 (Baduanjin):ti,ab,kw OR (Baduanjin qigong):ti,ab,kw  #5 (eight-section brocades):ti,ab,kw OR (eight-section exercises):ti,ab,kw OR (eight-treasured exercises):ti,ab,kw OR (baduanjin exercise):ti,ab,kw OR (baduanjin exercises):ti,ab,kw OR  #6 #4 OR #5 |  |
| 3 | Combined search | #3 AND #6 |  |
| 4 | Date | 1999 – January 31st, 2021 | **16** |
|  |  |  |  |
| **Table8**.Clarivate | | | |
| **Number** | **Search Terms** | **Search String** | **Hits:** |
| 1 | Hypertension | (((((((((((TS=(Hypertension)) OR TI=(Cardiovascular Disease)) OR TI=(cardiovascular diseases)) OR TI=(High Blood Pressures)) OR TI=(blood pressure)) OR TI=(blood pressures)) OR TI=(arterial pressure)) OR TI=(arterial pressures)) OR TI=(normotensive)) OR TI=(systolic pressure)) OR TI=(pulse pressure)) OR TI=(pre hypertension) |  |
| 2 | Baduanjin | (((((((TS=(baduanjin)) OR TI=(Baduanjin Qigong)) OR TI=(eight-section brocades))) OR TI=(eight-section exercises)) OR TS=(eight-treasured exercises)) OR TS=(baduanjin exercise)) OR TS=(baduanjin exercises) |  |
| 3 | Combined search | #1 AND #2 |  |
| 4 | Date | inception 1975 – January 31st, 2021 | **10** |
|  |  |  |  |
| **Table9**.ProQuest | | | |
| **Number** | **Search Terms** | **Search String** | **Hits:** |
| 1 | Hypertension | ((mainsubject(Hypertension) OR ti(Cardiovascular Disease) OR ti(cardiovascular diseases) OR ti(High Blood Pressures) OR ti(blood pressure) OR ti(blood pressures) OR ti(arterial pressure) OR ti(arterial pressures) OR ti(systolic pressure) OR ti(pulse pressure)) AND PEER(yes)) |  |
| 2 | Baduanjin | ((mainsubject(baduanjin) OR ti(Baduanjin Qigong) OR ti(eight-section brocades) OR ti(eight-section exercises) OR ti(eight-treasured exercises) OR ti(baduanjin exercise) OR ti(baduanjin exercises)) AND PEER(yes)) |  |
| 3 | Combined search | #1 AND #2 |  |
| 4 | Date | inception 1938 – January 31st, 2021 | 3 |
|  |  |  |  |
| **Table10**.MEDLINE | | | |
| **Number** | **Search Terms** | **Search String** | **Hits:** |
| 1 | Baduanjin | #6 #4 OR #5  #5 TI=(Baduanjin Qigong OR eight-section brocades OR eight-section exercises OR eight-treasured exercises OR baduanjin exercise OR baduanjin exercises)  #4 TS=(baduanin) |  |
| 2 | Hypertension | #3 #1 OR #2  #2 Tl=(Cardiovascular Disease OR cardiovascular diseases OR High Blood Pressures OR bloodpressure OR blood pressures OR arterial pressure OR arterial pressures OR normotensive OR systolic pressure OR pulse pressure OR pre hypertension)  #1 Ts=Hypertension |  |
| 3 | Combined search | #3 AND #6 |  |
| 4 | Date | inception 1966 – January 31st, 2021 | **7** |
|  |  |  |  |
| **Table11**.Scopus | | | |
| **Number** | **Search Terms** | **Search String** | **Hits:** |
| 1 | Baduanjin | ( TITLE ( baduanjin ) OR TITLE-ABS-KEY ( "baduanjin qigong" ) OR TITLE-ABS-KEY ( "eight-section brocades" ) OR TITLE-ABS-KEY ( "eight-section exercises" ) OR TITLE-ABS-KEY ( "eight-treasured exercises" ) OR TITLE-ABS-KEY ( "baduanjin exercise" ) OR TITLE-ABS-KEY ( "baduanjin exercises" ) ) |  |
| 2 | Hypertension | ( TITLE ( hypertension ) OR TITLE-ABS-KEY ( "Cardiovascular Diseases" ) OR TITLE-ABS-KEY ( "High Blood Pressures" ) OR TITLE-ABS-KEY ( "blood pressure" ) OR TITLE-ABS-KEY ( "blood pressures" ) OR TITLE-ABS-KEY ( "arterial pressure" ) OR TITLE-ABS-KEY ( "arterial pressures" ) OR TITLE-ABS-KEY ( "systolic pressure" ) OR TITLE-ABS-KEY ( "diastolic pressure" ) OR TITLE-ABS-KEY ( "pulse pressure" ) OR TITLE-ABS-KEY ( "venous pressure" ) OR TITLE-ABS-KEY ( "pressure monitor" ) OR TITLE-ABS-KEY ( "pre hypertension" ) OR TITLE-ABS-KEY ( "bp response" ) ) |  |
| 3 | Combined search | #1 AND #2 |  |
| 4 | Date | inception 1996 – January 31st, 2021 | **18** |
|  |  |  |  |
| **Table12**.CINAHL | | | |
| **Number** | **Search Terms** | **Search String** | **Hits:** |
| 1 | Hypertension | (((((((((((TS=(Hypertension)) OR TI=(Cardiovascular Disease)) OR TI=(cardiovascular diseases)) OR TI=(High Blood Pressures)) OR TI=(blood pressure)) OR TI=(blood pressures)) OR TI=(arterial pressure)) OR TI=(arterial pressures)) OR TI=(normotensive)) OR TI=(systolic pressure)) OR TI=(pulse pressure)) OR TI=(pre hypertension) |  |
| 2 | Baduanjin | (((((((TS=(baduanjin)) OR TI=(Baduanjin Qigong)) OR TI=(eight-section brocades))) OR TI=(eight-section exercises)) OR TS=(eight-treasured exercises)) OR TS=(baduanjin exercise)) OR TS=(baduanjin exercises) |  |
| 3 | Combined search | #1 AND #2 |  |
| 4 | Date | inception 1962 – January 31st, 2021 | **9** |
|  |  |  |  |

# 2 Supplementary Table 2.Stata Commands used in Meta-Analysis

| **Function/Analysis** | **Stata Commands** |
| --- | --- |
| Create new variable | gen |
| Study of heterogeneity | metan |
| Random-effect meta-analysis | metaan |
| Calculate mean effect sizes | meanes |
| Create funnel plots | metafunnel |
| Begg and Egger tests | metabias |
| meta regression | metareg |

# 3 Supplementary Table 3. Reference List of Included Baduanjin Trials

## PAN H，FENG Y(2010).Clinical Observation of Rehabilitation Therapy with Health Qigong Ba Duan Jin on Grade 1 Hypertension of Old Patients..[JOURNAL OF NANJING INSTITUTE OF PHYSICAL EDUCATION(NATURAL SCIENCE)](http://wf.nbsti.net/C/Periodical-njtyxyxb.aspx).[2010, 9(1)](http://wf.nbsti.net/C/periodical/njtyxyxb/2010-1.aspx). doi：[10.3969/j.issn.1671-5950.2010.01.002](http://dx.doi.org/10.3969/j.issn.1671-5950.2010.01.002)

## 2.CHEN W, LU Q,LIAN Y.(2020).Influence of the effect of seating Baduanjin exercise combined with health education on the antihypertensive effect of elderly hypertensive patients in the community[Shanghai Medical & Pharmaceutical Journal](http://wf.nbsti.net/C/Periodical-shyy.aspx).41(8).

3.Lian Y,Chen W,Lv Q;Wang YH,Wang Y.(2020).Effect of sitting Baduanjin Exercise on elderly hypertension with Yin deficiency and yang hyperactivity.[Chinese Journal of Integrative Medicine on Cardio/Cerebrovascular Disease](http://wf.nbsti.net/C/Periodical-zxyjhxnxgbzz.aspx).18(22).doi：[10.12102/j.issn.1672-1349.2020.22.032](http://dx.doi.org/10.12102/j.issn.1672-1349.2020.22.032)

4.HE X.(2015)Rehabilitation therapeutic effect of baduanjin training in aged patients with hypertension.[Chinese Journal of Cardiovascular Rehabilitation Medicine](http://wf.nbsti.net/C/Periodical-xxgkfyxzz.aspx).(3).doi：[10.3969/j.issn.1008-0074.2015.03.07](http://dx.doi.org/10.3969/j.issn.1008-0074.2015.03.07)

5.Jian Y,Huang X,Li H,Zhong L.(2020)Effect of antihypertensive Baduanjin Exercise Intervention on blood pressure and blood lipid in patients with essential hypertension.Journal of traditional Chinese Medicine.[(S02)](javascript:searchLink('','true','zh','@%E4%B8%AD%E5%8C%BB%E5%AD%A6%E6%8A%A5@[%E5%88%8A%E5%90%8D]%20%20%20AND%20@2020@[%E5%B9%B4]%20%20%20AND%20@S02@[%E6%9C%9F]');): 0145-0146.

6.Dong D,Yu ZD,Yu ZS.(2020)Intervention effect of fitness Qigong Baduanjin on hypertension patients with phlegm dampness stagnation.[Chinese Journal of Applied Physiology](http://wf.nbsti.net/C/Periodical-zgyyslxzz.aspx).36(2).doi：[10.12047/j.cjap.5924.2020.035](http://dx.doi.org/10.12047/j.cjap.5924.2020.035)

7.Lin Q,Yan X.(2017).Promoting effect of fitness Baduanjin on rehabilitation of elderly patients with hypertension.[Chinese Journal of Gerontology](http://wf.nbsti.net/C/Periodical-zglnxzz.aspx).[37(12)](http://wf.nbsti.net/C/periodical/zglnxzz/2017-12.aspx).doi：[10.3969/j.issn.1005-9202.2017.12.075](http://dx.doi.org/10.3969/j.issn.1005-9202.2017.12.075)

8.Chen Z.(2019)Mechanistic study of the effect of Baduanjin exercise intervention in essential hypertension based on the L-Arg / NOS / NO pathway[D].Fujian University of Traditional Chinese Medicine,(06).DOI:10.27021/d.cnki.gfjzc.2019.000056.

9.Chen Q.(2013).*Exploring the mechanism of Baduanjin on grade 1 hypertension from vascular endothelial function (*Master's dissertation, Fujian University of Traditional Chinese Medicine)

10.Chen L.(2016).Application of Baduanjin in Rehabilitation nursing of Elderly Hypertension patients.[Yiayao Qianyan](http://wf.nbsti.net/C/Periodical-yiyqy.aspx).6(22).

11.Liu Pi.(2014).*Clinical observation of Baduanjin exercise therapy for hypertension*(Master's thesis, Guangzhou University of Traditional Chinese Medicine)

12.Yu H.(2013).Clinical observation of 104 patients with hypertension and obesity. *The Chinese Clinician (*08),47-48.

13.Liang Y, Liao S, Han C, Wang Hong & Peng Ying.(2014). Effect of Baduanjin exercise intervention on blood pressure and blood lipids in patients with essential hypertension. *Henan traditional Chinese medicine*(12),2380-2381. doi:10.16367/j.issn.1003-5028.2014.12.102.

14.Cai H,Tu L.(2018)The application of Baduanjin health exercise in the nursing of hypertension patients.[Health Guide](http://wf.nbsti.net/C/Periodical-ysbjzn-x.aspx).(14).doi：[10.3969/j.issn.1006-6845.2018.14.138](http://dx.doi.org/10.3969/j.issn.1006-6845.2018.14.138)

# 15. Zhang P, Jia X, Zhang G, Zhang P, Li Z.(2020).Intervention effect of Baduanjin and health education on middle-aged and elderly patients with hypertension. *Henan Medical Research (*04),716-718.

# 16.Yan F, Shang Y, Ma L, Li D,Yao Z.(2016).Baduanjin joint Health education study on hypertension intervention in middle-aged and elderly people in the community. *Chongqing medicine*(06),795-796.

## 17.Yang M,Hang L.Yang L,Zhuang J,Lu Y.(2013)Research on the Intervention Effect on People with Borderline Hypertension in Community by Baduanjin and Health Education.[Chinese Manipulation & Rehabilitation Medicine](http://wf.nbsti.net/C/Periodical-amydy.aspx).(3)

18.Chen H, Zhou Anna.(2012).Effect of Baduanjin on blood pressure and serum hypersensitive C-reactive protein in patients with essential hypertension. *The Chinese Journal of Rehabilitation Medicine*(02),178-179.

19.Yang H.(2014).*Effect of Baduanjin on survival quality and autonomic response in hypertensive patients (*Master's dissertation, Hebei Union University)

20.Liang H, Huang C, Li D.(2016).Effect of Baduanjin on blood pressure and quality of life in patients with simple systolic hypertension.Massage and Rehabilitation Medicine(16),12-15.

21.Fan G,Li C.(2014).八段锦对1期老年高血压患者的血压及生活质量的影响.[Yiayao Qianyan](http://wf.nbsti.net/C/Periodical-yiyqy.aspx).(17)

22.Luo T.(2020).*Evaluation of Baduanjin efficacy in patients with grade 1 hypertension without target organ damage*(Master's thesis, Fujian University of Traditional Chinese Medicine).

## 23.Liu X.(2014).*Effect of Baduanjin intervention in patients with grade 1 and 2 essential hypertension*(Master's dissertation, Guangxi Normal University)

24. Lin F, He Q. (2014).Observation of Baduanjin exercise on grade 1 hypertension in the elderly. *Chinese Geriatric Health Care Medicine (0*3),25-26.

25.Yang H, Zhao Y & Li J. (2014).Effects of Baduanjin exercise on autonomic responses in hypertensive patients.*Chinese Medical Journal of the National Coal Industry (07), 1143-1146.*

26.Dong C & Zhang Y.(2016).Application of "antihypertensive Baduanjin" in blood pressure control in middle-aged patients with grade I essential hypertension. *The PLA Nursing Journal*(20),32-35.

27.Jiang Y, Fu G, Wang Q, Bao X, Liu X.(2019).Effects of Baduanjin on elderly patients with hypertension and anxiety.*Qilu Nursing magazine*(19),104-106.

28.[Chunmei X](https://pubmed.ncbi.nlm.nih.gov/?term=Xiao+C&cauthor_id=26782880), Yang, Y., & Zhuang, Y. (2016). Effect of Health Qigong Ba Duan Jin on Blood Pressure of Individuals with Essential Hypertension. *Journal of the American Geriatrics Society*, *64*(1), 211–213. https://doi.org/10.1111/jgs.13913

# 4 Supplementary Table 4. Study and Baduanjin intervention characteristics of included Baduanjin interventions (*n* = 28)

| **Intervention** | **BP- lowering mechanism** | | **Medication use** | | **PA** | | **Diet during intervention** | **Control group** |
| --- | --- | --- | --- | --- | --- | --- | --- | --- |
|  |  |  |  |  |  |  |  |  |
|  | **Proposed** | **Measured*** | **At baseline** | **During intervention** | **At baseline** | **PA level other than Baduanjin** |  |  |
| Pan 2010 | Reduce sympathetic excitability, reduce catecholamine concentration in the blood, and reduce peripheral vascular resistance | Blood lipid level（HDL-C、TG、TC）、Plasma insulin, fasting blood glucose | Thiazide diuretics, traditional Chinese medicine | maintained | NA | NA | NA | NA |
| Chen W 2020 | NA | NA | Conventional antihypertensive drugs | maintained | NA | NA | NA | NA |
| Lian 2020 | NA | Quality of life scale（SF-36）、BMI | Conventional antihypertensive drugs | maintained | NA | NA | NA | Health guidance、 routine daily walking |
| He 2015 | Reduce sympathetic nerve excitability, reduce the concentration of catecholamine in blood, raise serum NO concentration, and improve vascular endothelial function | NA | Conventional antihypertensive drugs | Maintained | Normal physical exercise | NA | Dietary guidance | NA |
| Jia 2020 | NA | HDL-C、LDL-C、TG、TC | Conventional antihypertensive drugs | Maintained | NA | NA | NA | Health guidance |
| Dong D 2020 | Regulated the oxidative stress levels and decreased ET-1 | TCM constitution score | Norvasc or Amlodipine besylate or Micardis(telmisartan) etc | Maintained | NA | NA | NA | NA |
| Lin Q 2017 | To inhibit the CRP secretion of inflammatory cytokines and reduce the expression levels of serum CRP | NO content, and ET-1 content | Norvasc、Micardis(telmisartan) | Maintained | NA | NA | Dietary guidance | Health guidance |
| Chen Z 2019 | The role of promoting NO synthesis | Arg-II expression, NOS activity, NOS protein expression, and NO levels | Lercanidipine 、losartan potassium | Maintained | NA | NA | NA | Routine treatment 、Health guidance |
| Chen Q 2013 | NA | Serum NO concentration and plasma ET-1 concentration | Norvasc or Amlodipine besylate or Micardis(telmisartan) etc | Maintained | NA | NA | Eat a low salt low fat digestible diet | Health guidance |
| Chen L 2016 | NA | NA | NA | NA | NA | Sit and exercise | NA | NA |
| Liu P 2014 | NA | HbAlc、TC、TG、LDL-C、HDL-C、BMI | NA | NA | NA | NA | NA | routine daily activities |
| Yu 2013 | NA | BMI、waist-to-hipratio | NA | NA | NA | NA | NA | Health guidance |
| Liang Y 2014 | Sympathetic drive is diminished, vagal tone increases, and increased NO release causes endothelium-dependent vasodilation | HDL-C、LDL-C、TG、TC | NA | NA | NA | NA | NA | routine daily walking |
| Cai H 2018 | NA | NA | Benazepril、Hydrochlorothiazide） | NA | NA | NA | Dietary guidance | Health guidance |
| Zhang 2020 | Improve vascular endothelial function | Quality of Life Scale (SF-36) | Follow the doctor's advice | Follow the doctor's advice | fitting exercise | NA | Dietary guidance | routine care、Health guidance |
| Cai Y 2016 | Improve vascular endothelial function | BMI、WHR、心率 | NA | NA | NA | NA | NA | Health guidance |
| Yang 2013 | NA | HDL-C、LDL-C、TG、TC | NA | NA | NA | NA | Dietary guidance | Health guidance |
| Chen H 2012 | High concentrations of serum CRP promote vascular endothelial cell proliferation | hs-CRP | nifedipine | Maintained | NA | NA | No change | routine daily walking |
| Yang 2014 | Reduce the sympathetic tone | Quality of Life Scale (SF-36) | Maintained | Maintained | NA | NA | NA | Health guidance |
| Liang H 2016 | Increase serum NO concentration and reduce plasma concentration | simple quality of life scale | Amlodipine Besylate Tablet | Maintained | NA | NA | NA | routine daily activities |
| Fan 2014 | NA | Quality of Life Scale (SF-36) | NA | NA | NA | NA | NA | Health guidance |
| Luo 2020 | NA | Borg subjective force score | NA | NA | NA | NA | NA | Health guidance |
| Liu X 2014 | Reduce the blood norepinephrine, epinephrine, endothelin, etc | TG、CHOL、UA | NA | NA | NA | NA | NA | NA |
| Lin F 2014 | NA | NA | Amlodipine Besylate Tablet | NA | NA | NA | reducing sodium intake, quitting smoking、alcohol | NA |
| Yang2 2014 | Reduce sympathetic activity, vagal activity increases, and the release of vasoconstrictor substances such as angiotensin decreases | modified Bruce, heart rate variability (HRV) | Conventional antihypertensive drugs | Maintained | NA | NA | Low-salt and low-fat levels | Health guidance |
| Dong C 2016 | Reduce sympathetic excitability; reduce peripheral vascular resistance by regulating the balance state of prostacyclin and catecholamines | NA | NA | NA | NA | NA | NA | Health guidance |
| Jiang 2019 | The imbalance state of cardiac autonomic nerve function is improved | TCM syndrome score and SAS score | Conventional antihypertensive drugs and anti-anxiety drugs | Maintained | NA | NA | reducing sodium intake, quitting smoking、alcohol | Health guidance |
| Chunmei, X 2016 | Reducdecreased plasma ET-1 levels in skeletal muscle and increased serum NO | HDL-C、serum NO、LDL-C、TC、Triglycerides、 fasting blood glucose,、and plasma ET-1 | NA | NA | NA | NA | NA | NA |

Note: *(Y)=results favoring Baduanjin intervention group were found in the measured mechanism, (N)= no results favoring Baduanjin intervention group were found in the measured mechanism.

Abbreviations: TCM= Traditional Chinese medical/Traditional Chinese medicine; PA = physical activity.

**5 Supplementary Table 5. Risk of bias for randomized controlled trials evaluated using the Cochrane Risk of Bias Tool (RoB 1)**

| **Study** | **Risk of Bias Judgement for Bias Rising from the/due to** | | | | | | | | **Overall rating** |
| --- | --- | --- | --- | --- | --- | --- | --- | --- | --- |
|  | Random sequence generation (selection bias) | | Allocation concealment (selection bias) | Blinding of participants and personnel (performance bias) | Blinding of outcome assessment (detection bias) | Incomplete outcome data (attrition bias) | selective reporting (reporting bias) | other bias |  |
| Pan 2010 | SC | SC | | SC | HR | SC | SC | SC | HR |
| Chen W 2020 | SC | SC | | SC | HR | SC | SC | SC | HR |
| Lian 2020 | SC | HR | | HR | HR | SC | SC | SC | HR |
| He 2015 | SC | SC | | SC | HR | SC | SC | SC | HR |
| Jia 2020 | SC | HR | | HR | HR | LR | SC | SC | HR |
| Dong D 2020 | SC | HR | | SC | LR | SC | SC | SC | HR |
| Lin Q 2017 | SC | HR | | SC | HR | SC | SC | SC | HR |
| Chen Z 2019 | SC | SC | | SC | SC | LR | SC | SC | SC |
| Chen Q 2013 | SC | SC | | LR | HR | LR | SC | SC | HR |
| Chen L 2016 | SC | SC | | SC | HR | SC | SC | SC | HR |
| Yu 2013 | SC | HR | | LR | HR | SC | SC | SC | HR |
| Liang Y 2014 | SC | SC | | SC | HR | SC | SC | SC | HR |
| Zhang 2020 | SC | HR | | HR | HR | SC | SC | SC | HR |
| Cai Y 2016 | SC | HR | | LR | HR | LR | SC | SC | HR |
| Yang 2013 | SC | HR | | LR | HR | SC | SC | SC | HR |
| Chen H 2012 | SC | HR | | LR | HR | SC | SC | SC | HR |
| Yang 2014 | SC | HR | | LR | HR | LR | SC | SC | HR |
| Liang H 2016 | SC | HR | | LR | HR | SC | SC | SC | HR |
| Fan 2014 | SC | HR | | HR | HR | SC | SC | SC | HR |
| Luo 2020 | SC | SC | | SC | HR | LR | SC | SC | HR |
| Liu X 2014 | SC | HR | | LR | HR | SC | LR | SC | HR |
| Lin F 2014 | SC | SC | | SC | HR | SC | SC | SC | HR |
| Yang2 2014 | SC | HR | | HR | HR | SC | SC | SC | HR |
| Dong C 2016 | SC | SC | | SC | HR | SC | SC | SC | HR |
| Jiang 2019 | SC | SC | | SC | HR | SC | SC | SC | HR |
| Chunmei, X 2016 | SC | HR | | LR | HR | SC | SC | SC | HR |

Note* Abbreviations: HR = high risk; LR = low risk; SC = some concerns.

Study:First author, last name and first letter of first name；publishing year

**6 Table 6. Risk of bias for non-randomized controlled trials evaluated using the methodological index for non-randomized studies (MINORS)**

| Study | **Risk of Bias Judgement for Bias Rising from the/due to** | | | | | | | | | | | | **Overall rating*** |
| --- | --- | --- | --- | --- | --- | --- | --- | --- | --- | --- | --- | --- | --- |
|  | A clearly stated aim | Inclusion of consecutive patients | Prospective collection of data | Endpoints appropriate to the aim of the study | Unbiased assessment of the study endpoint | Follow-up period appropriate to the aim of the study | Loss to follow up less than 5% | Prospective calculation of the study size | An adequate control group | Contemporary groups | Baseline equivalence of groups | Adequate statistical analyses |  |
| Liu P 2014 | 2 | 1 | 2 | 2 | 1 | 2 | 2 | 2 | 2 | 2 | 2 | 2 | 22 |
| Cai H 2018 | 2 | 1 | 2 | 2 | 1 | 2 | 2 | 2 | 2 | 2 | 2 | 2 | 22 |

Note* Abbreviations: 0 – 8 were low quality, 9 – 16 medium quality and 17 – 24 high quality.

Study:First author, last name and first letter of first name；publishing year

**7 Supplementary Table 7. Baseline sample characteristics of the Baduanjin and control intervention groups (*k =28)***

|  | ***k*** | **Baduanjin(*n* = 1061)** | **Control (*n* = 1060)** |
| --- | --- | --- | --- |
| **Age (year)** | 22 | 61.74±5.85 | 61.88± 6.14 |
| **Female (%)** | 24 | 51.31± 7.46 | 48.51± 8.15 |
| **Course**  **(year)** | 14 | 7.23± 4.20 | 7.45 ± 4.14 |
| **Baseline pulse pressure** | 28 | 57.54±8.47 | |
| **Baseline SBP (mmHg)** | 28 | 150.7±9.2 | 150.4±9.6 |
| **Baseline DBP (mmHg)** | 28 | 93.2±8.8 | 92.6±8.8 |
| Note* Statistics are summarized as mean ± SD;  Abbreviations: DBP = diastolic blood pressure; SBP = systolic blood pressure;Course =course of disease. | | | |

**8 Supplementary fig. 1, Funnel plot of SBP reduction in age subgroups**


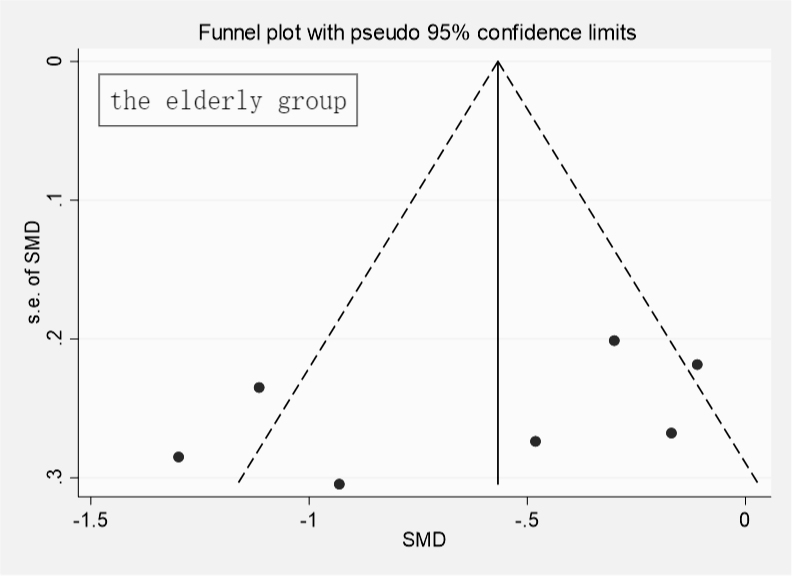

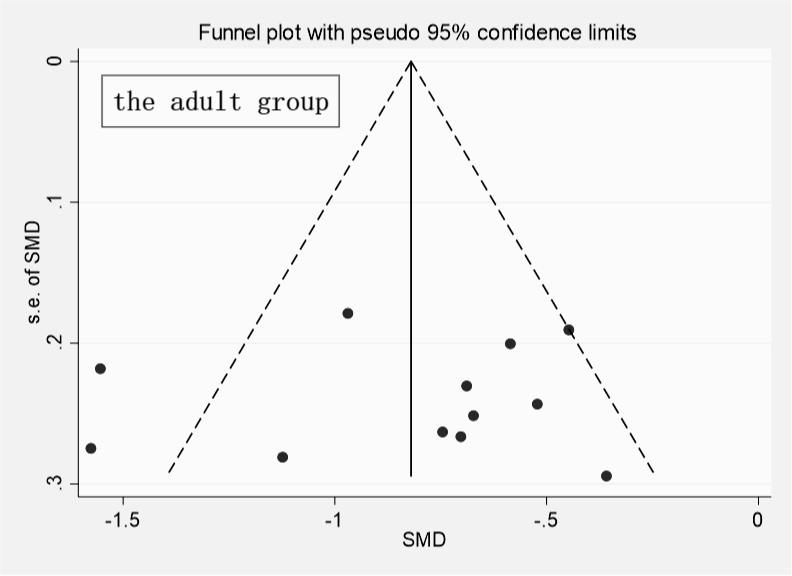


**9 Supplementary fig. 2, Funnel plot of SBP reduction in age subgroups**


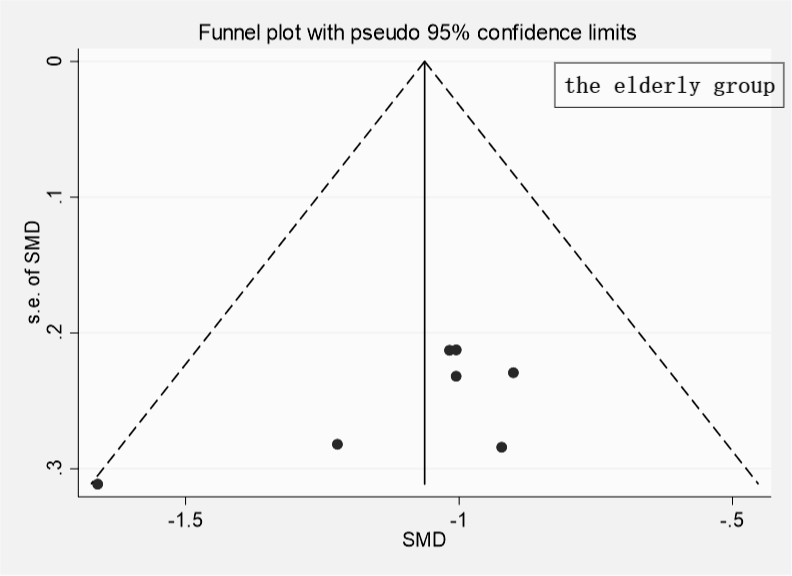

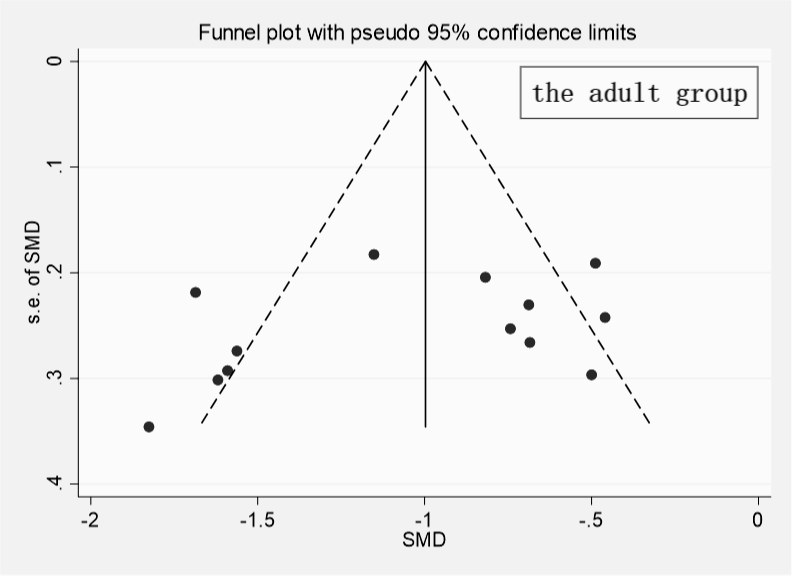

Supplement: Supplementary file 1 [file Data_Sheet_1.docx]
